# The Impact of Social Pressure and Monetary Incentive on Cognitive Control

**DOI:** 10.3389/fpsyg.2016.00093

**Published:** 2016-02-09

**Authors:** Mina Ličen, Frank Hartmann, Grega Repovš, Sergeja Slapničar

**Affiliations:** ^1^Department of Accounting and Auditing, Faculty of Economics, University of LjubljanaLjubljana, Slovenia; ^2^Department of Accounting and Control, Rotterdam School of Management, Erasmus University RotterdamRotterdam, Netherlands; ^3^Mind and Brain Lab, Department of Psychology, Faculty of Arts, University of LjubljanaLjubljana, Slovenia

**Keywords:** monetary incentives, social pressure, cognitive control, proactive index, AX-CPT

## Abstract

We compare the effects of two prominent organizational control mechanisms—social pressure and monetary incentive—on cognitive control. Cognitive control underlies the human ability to regulate thoughts and actions in the pursuit of behavioral goals. Previous studies show that monetary incentives can contribute to goal-oriented behavior by activating proactive control. There is, however, much less evidence of how social pressure affects cognitive control and task performance. In a within-subject experimental design, we tested 47 subjects performing the AX-CPT task to compare the activation of cognitive control modes under social pressure and monetary incentive beyond mere instructions to perform better. Our results indicate that instructing participants to improve their performance on its own leads to a significant shift from a reactive to a proactive control mode and that both social pressure and monetary incentive further enhance performance.

## Introduction

Organizations often implement formal control mechanisms to enhance the performance of their employees. Although such mechanisms can be designed in various ways, they all fundamentally rely on the use of positive and negative incentives, which are often monetary or social. Their positive effect on performance is thought to occur because they induce increased cognitive effort in employees (Curley et al., 1986; Trautmann et al., 2008; Vieider, 2009). However, the limited effectiveness of incentive-based organizational control systems is increasingly acknowledged in the management control literature. The reason may lie in the fact that control systems are predominantly designed to stimulate goal-oriented behavior with performance contingent incentives, whereas in a growing number of tasks the best performance is achieved by flexible adaptation to a changing environment. Performance contingent incentives may, for example, suppress learning and creativity at the individual level, thereby hampering social and economic innovation in organizations and society (Collins and Collins, 2002; Sen, 2008). Thus far, no simple control solution has been found to regulate this. Mainstream management control literature still associates human decisions that deviate from goal-oriented behavior, those that are flexible and influenced by emotions (Loewenstein and Lerner, 2003) with weaker impulse, emotional, and cognitive control.

This lack of understanding of the consequences of different incentives is in stark contrast with the trend to implement various mechanisms wherever they seem needed to control human behavior. To fully understand and to properly and effectively implement management control, further research on their effects on fundamental cognitive processes is needed. In this study, we focus on the ability to engage cognitive control and to change cognitive control strategies in response to monetary incentives and social pressure. Social pressure arises from mere identifiability of results and performance evaluation by others (Lerner and Tetlock, 1999), which in turn may create anxiety or a fear of failure. The effect of social pressure on performance is not straightforward. For example, Schmid et al. (2015) report that fear of public embarrassment and negative evaluation, born out of the need to perform well in front of others may, paradoxically, impair performance. On the other hand, transparent performance evaluation in organizations is found to positively affect performance (Latham and Locke, 2006). Given the ever-increasing amounts of money spent to incentivize managers, we were specifically interested in the effect of social pressure compared to monetary incentives on cognitive control and the enhancement of cognitive performance. While the effect of monetary incentives has been extensively documented in cognitive psychology (Locke and Braver, 2008; Braver et al., 2009; Dambacher et al., 2011; Padmala and Pessoa, 2011; Chiew and Braver, 2013, 2014; Fröber and Dreisbach, 2014), the effect of social pressure on performance in cognitive tasks has received little attention. To the best of our knowledge, these two types of incentives have not yet been directly compared within a single study. To account for their specific effects, we contrast them to the effects of mere instructions.

Cognitive control denotes the ability to manage one's own cognitive processes and is typically related to a number of processes such as working memory, reasoning, problem-solving, task flexibility, planning, and execution (Cohen et al., 1996; Braver and Barch, 2000; Botvinick et al., 2001; Miller and Cohen, 2001; Braver et al., 2002). It enables the regulation of thoughts and actions in pursuit of behavioral goals (Braver, 2012). It is essential in directing attention to a stimulus, shifting response strategies according to changes in the environment, and inhibiting more automatic or habitual response tendencies (Robertson et al., 2015). Cognitive control is exerted to supersede self-serving impulses and engage in socially desirable behavior (Pitesa et al., 2013). It is, hence, one of the most important determinants of efficient goal directed cognitive and behavioral performance.

According to the Dual Mechanism of Control theory (DMC; Braver et al., 2007), cognitive control operates through two distinct operating modes: proactive and reactive control (Braver and Barch, 2002; Braver et al., 2009; Braver, 2012). The proactive mode is future-oriented, helping to prepare the cognitive system for forthcoming events through the predictive use of current context. The processing of information occurs in a sustained, goal-oriented way. In contrast, reactive control is retrospective, backward-looking and reacts to the presence of urgent events by engaging control only if needed. Thus, the processing of information occurs in a more automatic, stimulus-driven, transient, and reflexive fashion (Braver, 2012). Whereas proactive control is based on anticipating and preparing for certain situations and events before they occur, reactive control is based on detecting conflict and implementing a response after its onset. Braver et al. (2007) hypothesize that a cognitive system's default mode is reactive control since it is usable in more situations and has lower demands on metabolic resources. In contrast, the proactive control mode is only temporarily invoked in more complex situations that demand more cognitive effort. DMC presents a useful framework for explaining dynamic shifts in the use of cognitive strategies in response to various stimuli.

Extensive research provides robust evidence that monetary incentives activate the proactive control mode (Locke and Braver, 2008; Braver et al., 2009; Engelmann et al., 2009; Jimura et al., 2010; Padmala and Pessoa, 2011; Chiew and Braver, 2013, 2014; Fröber and Dreisbach, 2014). Neuroscientific studies suggest that proactive and reactive control can be clearly distinguished in the activity of different brain regions (Braver et al., 2007, 2009). Proactive control, directed to reward maximization, is associated with increased sustained activity in the dorsolateral prefrontal cortex (DLPFC), which is believed to be central in actively maintaining goals and instructions. It influences information processing in other brain regions in line with maintained information. The DLPFC is interconnected with the midbrain DA system, the anterior cingulate cortex (ACC) and the medial temporal lobe complex. The constant firing of the DA system that signals reward-related salience of predictive cues ensures a sustained activity of the DLPFC. Reactive control, on the other hand, is not oriented to maximizing rewards but to resolving interference. The dorsal ACC (dACC) is associated with conflict monitoring. When detecting a response conflict or an impending error, it rapidly signals the need for increased control to the DLPFC on the current trial and is only transiently activated (Sawaguchi et al., 1988; Sawaguchi and Goldman-Rakic, 1991; Arnsten et al., 1994; Schmid et al., 2015).

There is contradictory evidence of how social pressure contributes to goal-oriented cognition and behavior. The identifiability of results is known to be influential in directing agents' behavior. As argued by Lerner and Tetlock (1999), the mere presence of a superior and knowledge of his/her expectations elicits conforming behavior. Moreover, setting ambitious goals, which are accepted by the employee, can be a powerful driver of performance improvement (Latham and Locke, 2006). People's natural tendency to behave in conformity with the expectations of those they are accountable to, albeit without an explicit monetary incentive, has been documented by a large body of research (Cialdini et al., 1976; Tetlock, 1983; Tetlock et al., 1989; Klimoski and Inks, 1990; Quinn and Schlenker, 2002). The reason for such behavior may lie in a broader definition of motivation according to which both the anticipation of a possible reward or the avoidance of sanctions facilitate behavior (Taylor et al., 2004).

According to other accounts (e.g., Hickman and Metz, 2015; Schmid et al., 2015) the transparency of results and their identifiability may be perceived as a socially threatening stimulus, which requires conflict processing during task execution. Schmid et al. (2015) report that individuals who are more sensitive to such pressures rely more heavily on a reactive control strategy driven by conflict-processing dACC activity and that socially anxious individuals show poorer performance compared to less anxious ones in cognitive tasks that require goal-directed behavior. Hickman and Metz (2015) analyzed a phenomenon common in sport, but also in other contexts, whereby large rewards and expectations create such psychological pressure that the performance eventually worsens (known as choking). They recognize that the intertwining effects of explicit monitoring and high rewards are difficult to disentangle, but after controlling for them, they provide evidence of a negative relationship between the size of the reward and the performance.

To investigate how social pressure influences cognitive control modes and cognitive performance compared to monetary incentive beyond simple instructions to perform better, we employed a within-subjects experimental design in which 47 students performed the AX-Continuous Performance Task (AX-CPT; Cohen and Servan-Schreiber, 1992; Servan-Schreiber et al., 1996; Braver et al., 2001) under social pressure, monetary incentive, and a control condition (instructions only). This cognitive task measures goal representation, maintenance, and information updates, and has often been used to examine underlying modes of cognitive control (Barch et al., 1997; Braver et al., 2001, 2005; Braver and Bongiolatti, 2002; McDonald and Carter, 2003; Paxton et al., 2006, 2008; Locke and Braver, 2008). Due to conflicting evidence regarding social pressure on cognitive performance, we did not have strong *a priori* predictions related to its effect on cognitive control strategy and performance.

## Materials and methods

### Participants and task

Fifty-six undergraduate students (age *M* = 21.96, *SD* = 1.76, range = 18–27 years; 19 male) majoring in Accounting, Management, and Finance at the Faculty of Economics at the University of Ljubljana took part in the study. Their work experience ranged from 0 to 9 years (*M* = 2.95, *SD* = 2.36). Participants were invited to participate in the study as an opportunity to earn course credits. Linked to their task performance, students could also earn monetary reward ranging between EUR 0 and EUR 6. The average amount earned was EUR 3.7 for 45 min of activity, which approximately corresponds to the hourly rate for student work. All participants were informed that their participation was voluntary and that they were free to withdraw from the study at any point. They gave written informed consent prior to participation in the study. The study and the procedures followed were in accordance with the Helsinki Declaration as revised in 2013.

Nine participants (16.07%) were eliminated from further analysis due to the lack of correct responses in the AY trials in one or more manipulated incentive conditions^1^. Our final analysis included 47 participants (16 male, age *M* = 21.92, *SD* = 1.84, range = 18–27 years; work experience *M* = 2.92, *SD* = 2.27, range = 0–9 years).

We used the AX-CPT task developed to measure proactive and reactive control modes (Braver, 2012). The task was the following: Pairs of letters were displayed sequentially on a computer screen. The first letter, either A or B, appeared as a cue. The second letter, either X or Y, was considered the probe. In combination, there were four types of trials, i.e., AX, AY, BX, and BY trials (Kam et al., 2012). The participants' task was to respond as quickly and accurately as possible to each probe following the cue. Specifically, participants had to press the letter N with their index finger when A was followed by a target probe X (target trial). The three other trial types were non-target trials in which A was followed by Y, or B was followed by either Y or X (AY, BY, and BX). In the case of a non-target trial, participants had to press the letter M with their middle finger. In the calibration phase, participants performed a block of 30 trials to calibrate the criterion for the response speed for each individual. Then, four blocks of 30 trials were presented for each of the three different incentive conditions. Thus, overall the participants performed 390 trials. Within each block, the most common trial presented was the AX target trial, which occurred in 70% of the trials, with the remaining 30% equally distributed among the other three non-target trials (AY, BX, and BY). These pairs were presented in random order. After each trial, feedback was given to participants informing them whether their answer was correct and fast enough (see Section Procedure and Manipulation).

Because the target trial AX occurs with a high frequency, it not only creates a strong preparatory attentional expectancy triggered by the contextual cues (A = target; B = non-target), but also creates a target response bias linked to the X probe. Thus, the utilization of proactive control can be indexed on AY trials (Chiew and Braver, 2013). The AY trial type requires inhibition of a prepotent response, as a subject is primed to make an incorrect target response as soon as they see cue A (Kam et al., 2012). Thus, the AY trial type requires subjects to alter their usual action plans and press the letter “M” instead of the letter “N”. According to Chiew and Braver (2013), stronger interference in these trials (in terms of greater errors and slower reaction times) can be interpreted as the activation of strong proactive control. Alternatively, reactive control can be indexed on BX trials. The BX trial type requires subjects to actively maintain a representation of the context provided by the cue stimulus in their working memory in order to press the correct button when seeing probe X (Kam et al., 2012). In this condition, a subject has to keep in mind that cue B, and not A, was shown before X. Stronger interference in these trials (in terms of greater errors and slower reaction times) can be interpreted as the engagement of reactive control/lack of proactive control (Chiew and Braver, 2013). Relative performance in AY vs. BX trials therefore provides an indication of whether proactive or reactive control is dominant (Kam et al., 2012; Chiew and Braver, 2013; Lamm et al., 2013).

The AX-CPT task was programmed in the E-prime 2.0 software running on Windows 7 OS. The stimuli were presented on a 19-inch LCD display. To familiarize themselves with the task, participants first performed 10 practice trials during which their performance was not recorded.

**Figure 1:**
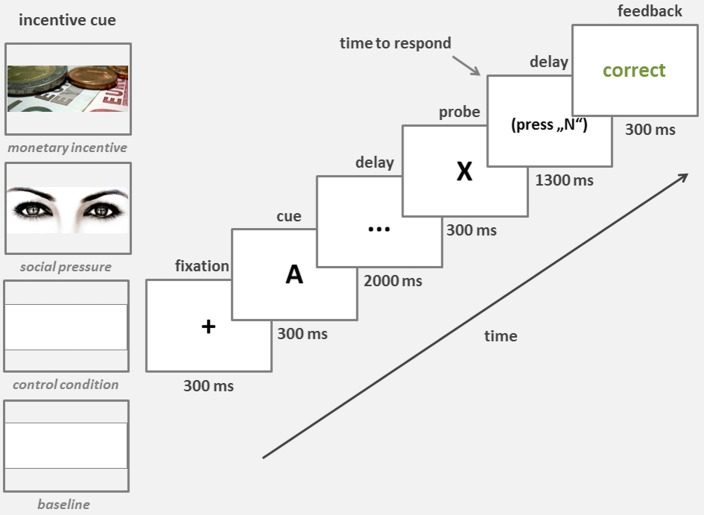
**Trial structure and timing**. An example of an AX (target) trial is shown with both a non-incentive (baseline and control condition—no picture) and an incentive (monetary incentive—a picture of money; social pressure—a picture of eyes) cues. One of these three cues was shown on the screen during each condition block, indicating the incentive type.

Performance feedback was provided after each trial. It was presented for 300 milliseconds (ms). Trials lasted 4.5 s and consisted of the following sequence of events (see Figure 1): fixation (300 ms), cue (300 ms), delay 1 (2000 ms), probe (300 ms), delay 2 (1300 ms), and feedback (300 ms).

### Procedure and manipulation

To individually calibrate the criterion speed of response to the probe stimuli in the incentive conditions, the procedure started with a baseline condition in which participants were told to perform the tasks as fast and as accurately as possible. Subjects performed the baseline block without any knowledge of the subsequent procedure and incentives. We then introduced three conditions in randomly interchangeable blocks. In all three conditions, participants were asked to improve their speed of response while still responding correctly. Participants in the monetary incentive block, which was cued by a picture of money (see Figure 1), were told they could earn EUR 0.05 for each fast and correct response, but could be penalized with a deduction of EUR 0.05 for each incorrect response. The speed criterion was set so that participants had to match or exceed the speed of the fastest third of all responses in the baseline condition. In the social pressure condition, participants were told that their results and ranking within the group of participants would be publicly announced. This block started with a picture of eyes on the screen (see Figure 1). The control block started with no cue. This condition was introduced to account for the effect of mere instructions.

The experimental design was block-based with counterbalanced order of condition blocks across participants. The advantage of a block-based experimental design is that it allows the examination of a sustained motivational effect on cognitive control dynamics rather than a transient effect (Chiew and Braver, 2013; Lamm et al., 2013). The experiment was conducted in small groups of 16 participants in a quiet, well-lit room. After the task was completed, participants filled out a demographics questionnaire and were informed about how much money they had earned.

### Data analysis

To eliminate automatic button pressing and extreme outliers from the analysis, we excluded responses faster than 200^2^ ms and slower than 2000 ms (2.26%; Schouppe et al., 2015) and all incorrect responses^3^ (0.04%). In addition, responses that deviated by more than two standard deviations from the conditions' mean were removed from reaction time analyses as outliers (4.20%; Lamm et al., 2013). We tested for and found no significant effect of age on performance. Task performance was measured and expressed as the mean reaction time for correct responses, mean error rate (percentage incorrect), and mean percent of fast responses. The responses were considered fast enough and correct if they matched or exceeded the speed of the fastest third of all responses in the baseline condition. To estimate the statistical significance of the behavioral performance data, we performed a repeated measures analysis of variance (ANOVA). In cases where the assumption of sphericity was violated, indicating that the variance of the differences between all combinations of related groups was not equal, we adjusted the degrees of freedom using the Greenhouse-Geisser correction method (Field, 2009).

To examine the underlying mode of cognitive control under manipulations, we performed an additional analysis on a direct measure of a cognitive control shift—a so-called proactive index (Braver et al., 2009). The index was computed from reaction times and error rates in the AY and BX trials as (AY − BX)/(AY + BX) and measures the relative tendency for proactive control (Braver et al., 2009). The proactive index calculation yields a score between −1 and +1: the closer the score is to +1, the more proactive is the cognitive control (Braver et al., 2009; Chiew and Braver, 2014). Namely, if subjects are more alert to the preceding cue and prepare their responses proactively, they will find it harder to inhibit the inappropriate response in the AY trials and will be even faster/make fewer errors in the BX trials, both leading to a higher value of the proactive index. As in some cases participants performed at ceiling, which resulted in a zero error rate, we used a corrected error rate, computed using the formula: (number of errors + 0.5)/(number of trials + 1) (Hautus, 1995).

## Results

The effect of the manipulations on task performance may be expressed in terms of improved reaction time and/or accuracy. We analyzed both, as well as any possible trade-off between them.

### Global incentive effect

Under all incentive conditions, participants were asked to improve the speed of their responses while still responding correctly. The cut-off for correct and fast enough responses was calculated for each participant as the speed of the upper third correct responses for all trial types in the baseline condition. There were 64.3% of such responses in the control condition, 67.6% in the social pressure condition, and 68.3% in the monetary incentive condition vs. the expected rate of 33.3%, had the performance remained at the baseline level. Similar results were reported by Chiew and Braver (2013, 2014), but only for the monetary incentive condition. Overall, the results show that the incentive manipulation was successful in improving performance. In accordance with the given instructions, participants achieved a higher rate of fast and correct responses in the control condition and in both incentive conditions.

### Baseline vs. control condition

**Figure 2:**
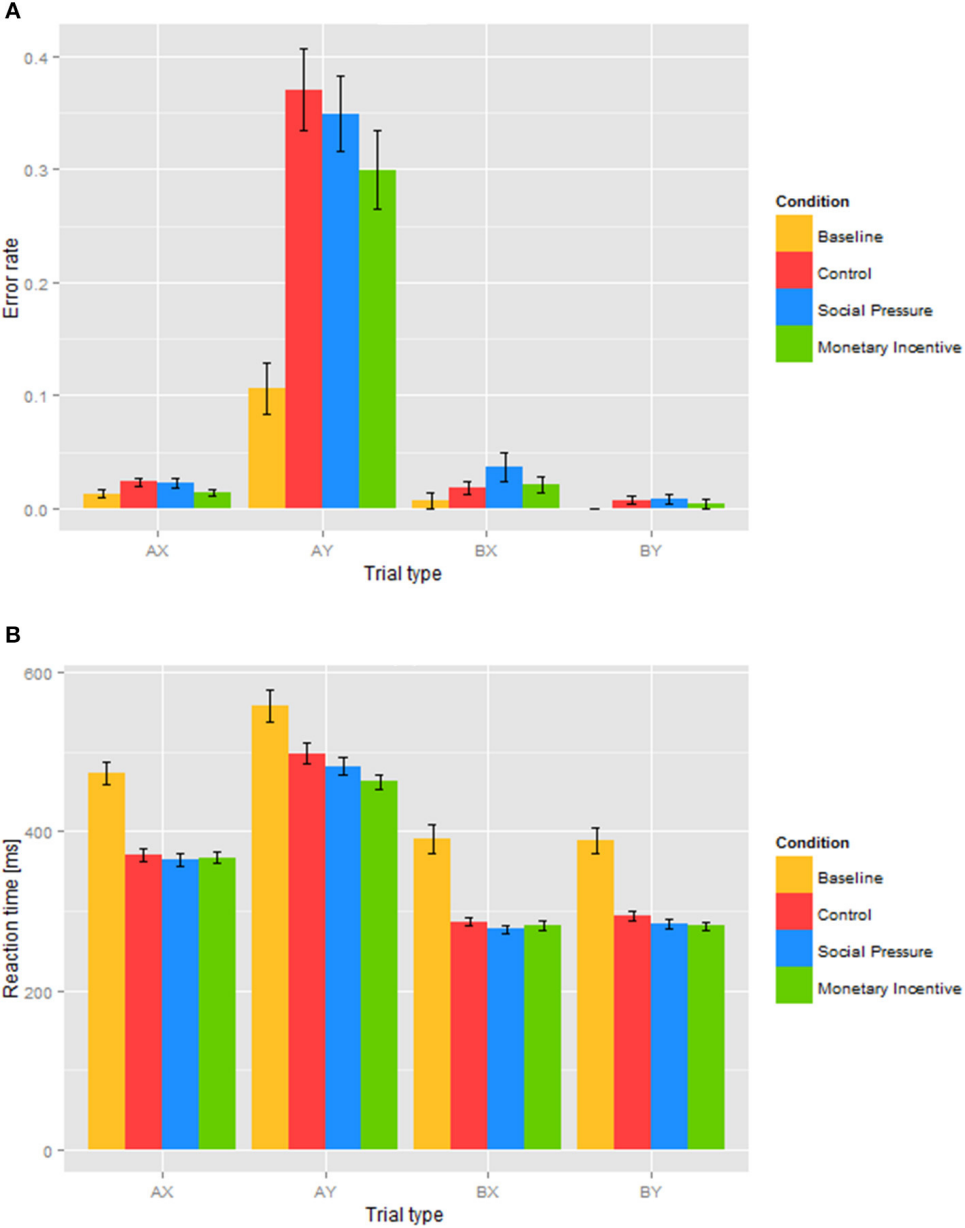
**Error rates (A) and reaction times (B) in different trial types and conditions**. Error rates **(A)** and reaction times of correct responses **(B)** for each of the trial types (AX, AY, BX, and BY) are shown for each condition.

To further assess the effect of the experimental instructions on task performance, we conducted a two-way repeated measures ANOVA with the within-subject factors condition (baseline, control) and trial type (AX, AY, BX, BY). The analysis of *error rates* showed a significant main effect of the condition, *F*_(1, 46)_ = 54.7, *p* < 0.001, reflecting an overall increase in error rates in the control condition (see Figure 2A), a significant main effect of trial type, *F*_(1.08, 49.5)_ = 81.7, *p* < 0.001, reflecting larger error rates in AY trials compared to AX, BY, and BX trials, and a significant condition × trial type interaction, *F*_(1.19, 54.6)_ = 48.6, *p* < 0.001, reflecting a larger increase in error rates from the baseline to the control condition in AY trials compared to AX, BY, and BX trials. To formally test the observed pattern of results, we conducted follow-up paired *t*-tests that showed significant increases in the control vs. the baseline condition error rates for AX, *t*_(46)_ = 2.16, *p* = 0.036, AY, *t*_(46)_ = 7.41, *p* < 0.001, and BY, *t*_(46)_ = 2.07, *p* = 0.044, but not BX, *t*_(46)_ = 1.17, *p* = 0.249, trials. Furthermore, comparing the extent of increases between AY and other trial types using paired *t*-tests revealed significantly higher increases in error rates from the baseline to the control condition for AY trials compared to AX, *t*_(46)_ = 7.13, *p* < 0.001, BX, *t*_(46)_ = 7.30, *p* < 0.001, and BY, *t*_(46)_ = 7.19, *p* < 0.001, trials, suggesting increased reliance on proactive control. No other comparison in the extent of increases between different trial types revealed significant differences (all *p* > 0.65).

The analysis of *reaction times* also revealed a significant main effect of the condition, *F*_(1, 46)_ = 55.3, *p* < 0.001, reflecting an overall reduction in reaction times from the baseline to the control condition (see Figure 2B), trial type, *F*_(2.05, 94.3)_ = 179.2, *p* < 0.001, reflecting longer reaction times in AX and AY compared to BX and BY trials, and a significant condition × trial type interaction, *F*_(2.33, 107.3)_ = 3.2, *p* = 0.037, reflecting larger reductions in reaction times in AX, BX, and BY than in AY trials. A pairwise comparison of the extent of reaction time reduction between the different trial types revealed significantly smaller decreases in reaction times from the baseline to the control condition in AY compared to AX, *t*_(46)_ = 3.09, *p* = 0.003, and BX, *t*_(46)_ = 2.12, *p* = 0.039, but not BY, *t*_(46)_ = 1.82, *p* = 0.074, trials, again suggesting increased reliance on proactive control. Significant improvement in reaction times in the control condition relative to the baseline may be interpreted as conformance to instructions, even in the absence of a direct incentive.

### Practice effect at baseline

Due to the within-subject block design of the study, there is a potential concern that differences between blocks reflect practice effects rather than experimental manipulations. To address this concern, we conducted a logistic regression on error rates and a linear regression analysis on reaction times in the baseline condition to test for the presence of performance improvement with the task progression reflecting the practice effect. The analysis was only conducted on the AX trials since they occurred in 70% of the trials. As the trials were manipulated within-subjects, the subjects were treated as a random factor and intercepts were modeled for each subject separately. Statistical significance was estimated using likelihood ratio χ^2^ tests.

Results of *logistic regression* of response accuracy (0 for an incorrect and 1 for a correct response) on a trial number revealed a significant positive effect, β = 0.08, $\chi_{\left(1\right)}^{2}=5.36$, *R*^2^ = 0.04, *p* = 0.021, suggesting that subjects improved their accuracy within the baseline block. However, as on average subjects only made 1.32% of errors in the baseline condition and mostly performed at ceiling, we conclude that differences in performance cannot be attributed to practice effects. Results of a *linear regression* of reaction times on a trial number revealed a non-significant positive effect, β = 0.18, $\chi_{\left(1\right)}^{2}=$ 0.28, *R*^2^ = 0.54, *p* = 0.598, suggesting the absence of the practice effect on reaction times in the baseline condition.

We extended the analysis of practice effects to the experimental blocks, but did not find any significant effect on accuracy and reaction times in any of the experimental conditions. We did find a small effect of fatigue reflected in reaction times in the social pressure condition (for detailed results see Supplementary Figure 1).

Taken together, no or minimal practice effect in the baseline condition and no evidence of practice effect in the experimental conditions reduces the possibility that the observed differences between experimental conditions reflect practice effect. As the purpose of the baseline condition was primarily to ascertain individual criterion reaction time, all further analyses focus on direct comparisons between the three experimental conditions.

### Comparison of experimental conditions

To investigate the effect of experimental conditions on error rates and reaction times, we conducted a two-way repeated measures ANOVA with the within-subject factors condition (control, social pressure, monetary incentive) and trial type (AX, AY, BX, BY). The analysis of *error rates* showed an expected significant main effect of trial type, *F*_(1.06, 48.9)_ = 121.2, *p* < 0.001, revealing significantly higher error rates in AY trials (see Figure 2A). Results also showed a significant main effect of the condition, *F*_(2, 92)_ = 3.6, *p* = 0.030. A follow- up paired *t*-test between conditions collapsed over all trial types revealed significantly lower error rates in the monetary incentive condition compared to both the control, *t*_(46)_ = 2.26, *p* = 0.029, and the social pressure conditions, *t*_(46)_ = 2.54, *p* = 0.015, but no significant differences between the social pressure and the control conditions, *t*_(46)_ = 0.09, *p* = 0.932. Though the pattern of error rates differed across trial types and seemed to be driven mostly by differences in AY trials, the absence of a significant condition × trial type interaction, *F*_(2.20, 101.2)_ = 2.4, *p* = 0.096, did not warrant further post-hoc exploration. These results suggest that the monetary incentive was the most successful in reducing error rates.

The analysis of *reaction times* also showed an expected significant main effect of trial type, *F*_(1.87, 86.2)_ = 452.6, *p* < 0.001, reflecting the shortest reaction times in BX and BY trials, somewhat longer reaction times in AX trials, and the longest reaction times in AY trials (see Figure 2B). ANOVA revealed a significant main effect of the condition, *F*_(2, 92)_ = 7.7, *p* < 0.001. Post hoc paired *t*-tests collapsed over trial types revealed significantly shorter reaction times in both the social pressure, *t*_(46)_ = 2.46, *p* = 0.018, and the monetary incentive condition, *t*_(46)_ = 3.74, *p* < 0.001, compared to the control condition, and no significant differences between the social pressure and monetary incentive conditions, *t*_(46)_ = 1.23, *p* = 0.226. The presence of a significant condition × trial type interaction, *F*_(3.19, 146.7)_ = 3.5, *p* = 0.015, warranted further investigation of experimental manipulation on each of the trial types by means of follow-up one-way repeated measures ANOVAs with factor condition (control, social pressure, monetary incentive). These revealed a significant effect of the condition in AY, *F*_(2, 92)_ = 6.4, *p* = 0.003, and BY trials, *F*_(2, 92)_ = 4.3, *p* = 0.017, but not in AX, *F*_(2, 92)_ = 0.97, *p* = 0.382, or BX trials, *F*_(2, 92)_ = 2.3, *p* = 0.110. Additional pairwise *t*-tests showed that reaction times in AY trials in the monetary incentive condition were significantly shorter than in the control condition, *t*_(46)_ = 3.46, *p* = 0.001, and in the social pressure condition, *t*_(46)_ = 2.24, *p* = 0.030. Reaction times in BY trials under monetary incentive were again significantly shorter than in the control condition, *t*_(46)_ = 2.90, *p* = 0.006, whereas the differences with the social pressure condition were not significant, *t*_(46)_ = 0.732, *p* = 0.468. The reduction in reaction times under social pressure compared to the control condition approached statistical significance, *t*_(46)_ = 1.96, *p* = 0.056. These findings indicate that while monetary incentive led to the strongest reduction in reaction times, social pressure had a similar effect in stimulating performance. In line with prior studies (Kam et al., 2012; Lamm et al., 2013; Chiew and Braver, 2014), participants had the slowest reaction time in AY trials compared to AX, BX, and BY trials, indicating the prevalence of proactive cognitive control.

### Speed-accuracy trade-off effect

To exclude the possibility that the observed changes in performance were due to a speed-accuracy trade-off rather than an actual performance improvement, we conducted a correlation analysis between the *change in error rates* and the *change in reaction times* in the baseline vs. the incentive conditions. This was of a particular concern since we observed higher speed, but also higher error rates under the experimental conditions. The results show a non-significant negative correlation between changes in speed and error rates in the monetary incentive, *r* = −0.02, *p* = 0.808, a non-significant positive correlation in the control condition, *r* = 0.12, *p* = 0.107, and a small but significant positive correlation under social pressure, *r* = 0.15, *p* = 0.036, indicating that subjects in the social pressure condition managed to improve both accuracy and reaction times (see Supplementary Figure 2). We can therefore dismiss the concern that subjects achieved a faster reaction time on account of significantly higher error rates. On the contrary, participants improved their response speed without any significant loss of accuracy, arguably by shifting to the proactive control mode.

### Correlation between incentive conditions

**Figure 3:**
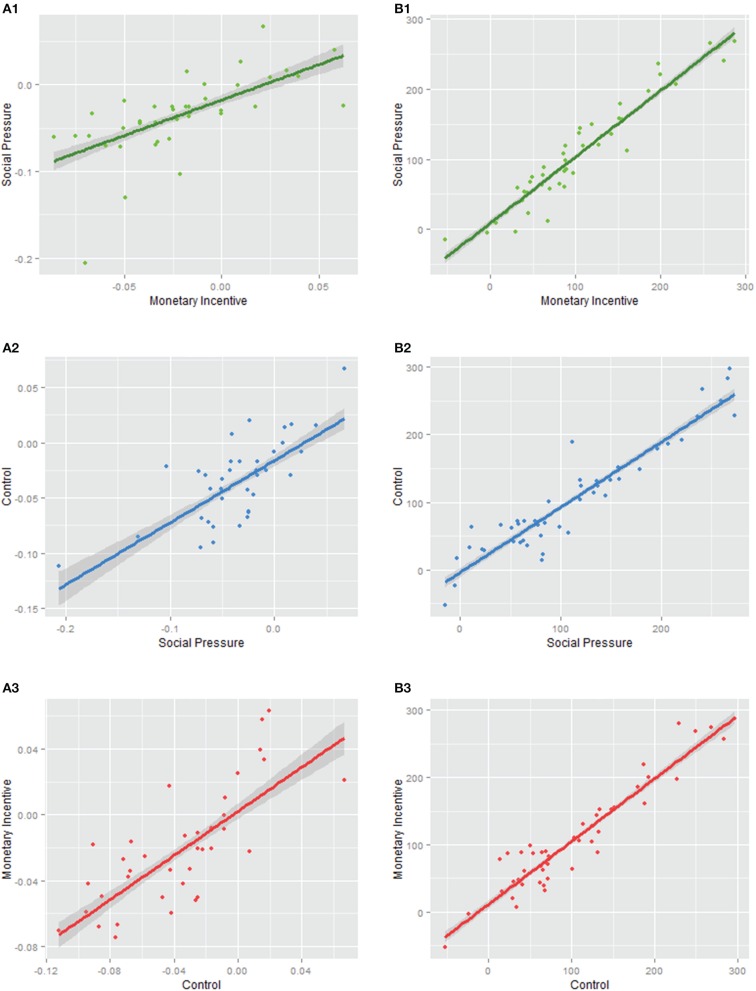
**Correlation between experimental conditions in the performance change relative to baseline as measured by error rates (A) and reaction times (B)**. Performance in terms of error rate **(A)** and reaction time of correct responses **(B)** under experimental conditions relative to baseline collapsed over the trial type are plotted between the monetary incentive and social pressure conditions (1), between the social pressure and control condition (2), and between the control and monetary incentive conditions (3). A positive correlation indicates that subjects were equally sensitive to both compared incentive conditions.

Some people are more stimulated, have greater motivational orientation and better cognitive resources than others. They might be equally sensitive to monetary and social incentives, while others are less sensitive to both. To analyze this issue, we conducted a correlation analysis between the differences among error rates and reaction times relative to baseline in incentive conditions. The results show strong and significant positive correlations between error rates and reaction times, respectively, across all pairs of comparisons; between monetary incentive and social pressure, *r* = 0.63, *p* < 0.001, *r* = 0.96, *p* < 0.001, the control condition and monetary incentive, *r* = 0.72, *p* < 0.001, *r* = 0.95, *p* < 0.001, and between the control condition and social pressure, *r* = 0.68, *p* < 0.001, *r* = 0.94, *p* < 0.001 (see Figure 3). The high degree of correlation between effects in the different incentive conditions suggests that the subjects were similarly sensitive to the incentives.

### Analysis of the proactive index

**Figure 4:**
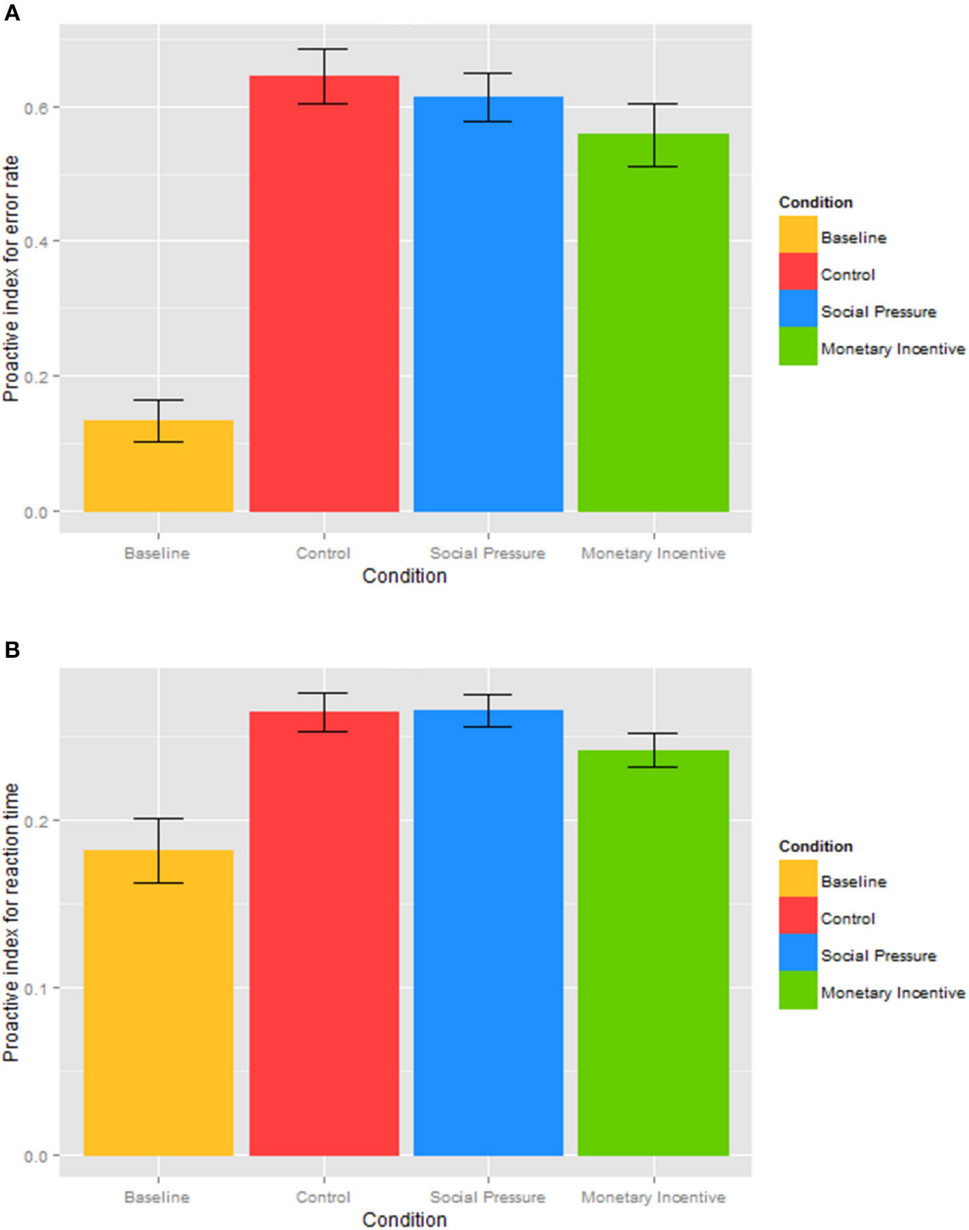
**Proactive index for the error rate (A) and reaction time (B) in different conditions**. The proactive index based on error rates **(A)** and reaction times **(B)** is shown for each of the incentive conditions: baseline, control, social pressure, and monetary incentive. The closer the score of the proactive index is to +1, the more proactive is the cognitive control.

To specifically focus on the possible effect of the experimental conditions on cognitive control, we analyzed a proactive index (Braver et al., 2009), computed for error rates (Figure 4A) and reaction times (Figure 4B). To examine the effect of the experimental conditions on the prevailing cognitive control strategy, we conducted a one-way repeated measures ANOVA with a within-subject factor condition (baseline, control, social pressure, monetary incentive). Results of the *proactive index for error rates* showed a significant main effect of the condition, *F*_(3, 138)_ = 49.5, *p* < 0.001, reflecting substantial increases in proactive control in all three conditions compared to the baseline. Post-hoc paired *t*-test indicated significantly higher value of the proactive index for error rates in the control, *t*_(46)_ = 11.3, *p* < 0.001, social pressure, *t*_(46)_ = 13.1, *p* < 0.001, and monetary incentive conditions, *t*_(46)_ = 8.6, *p* < 0.001, compared to the baseline. When comparing the three experimental conditions, a slight reduction in the proactive index in the monetary incentive condition compared to the control condition was not found to be significant, *t*_(46)_ = 1.7, *p* = 0.095, neither were all other comparisons (all *p* > 0.30).

Similarly, results of the *proactive index for reaction times* showed a significant effect of the condition, *F*_(1.96, 90.2)_ = 11.2, *p* < 0.001, again reflecting substantial increases in the proactive index in the control, *t*_(46)_ = 3.9, *p* < 0.001, social pressure, *t*_(46)_ = 4.2, *p* < 0.001, and monetary incentive conditions, *t*_(46)_ = 3.0, *p* = 0.004, compared to the baseline, indicating a shift to proactive control in all three manipulated conditions. Paired comparisons between the experimental conditions revealed a significantly lower value of the proactive index in the monetary incentive condition compared to the social pressure condition, *t*_(46)_ = 2.5, *p* = 0.015. The difference between the monetary incentive and the control condition approached significance, *t*_(46)_ = 1.8, *p* = 0.080, whereas the value of the proactive index did not differ between the social pressure and the control condition, *t*_(46)_ = 0.1, *p* = 0.941. This indicates that social pressure and mere instructions shift cognitive control to a stronger proactive mode than monetary incentive. The lower proactive indices under the monetary incentive condition could be attributed to the possibility of being penalized for incorrect responses, which forces an individual to make fewer errors and have a slower reaction time in the AY trials compared to the social pressure and control conditions.

**Figure 5:**
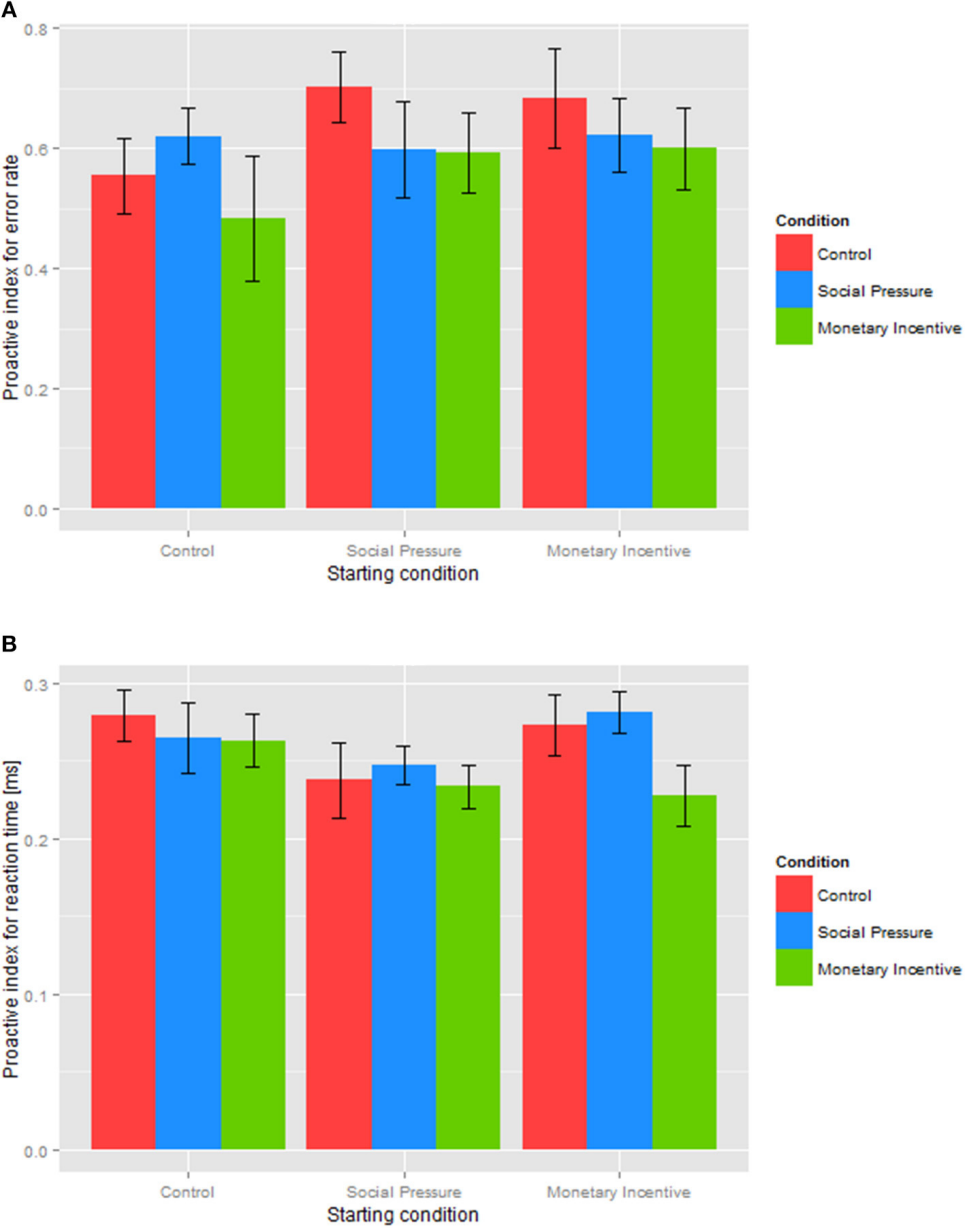
**The effect of the experimental condition order on the proactive index for error rates (A) and reaction times (B) in different conditions**. The proactive index for error rates **(A)** and reaction times **(B)** is shown for each of the incentive conditions: control, social pressure, and monetary incentive depending on the starting condition as a between-factor.

A possible concern in the presented analysis is that a change in cognitive control in one condition might persist in subsequent blocks, confounding the observed results. As the order of the experimental conditions was counterbalanced across participants, we were able to address this concern directly. We conducted a two-way repeated measures ANOVA with the within-subject factors condition (control, social pressure, monetary incentive) and a between-subject factor starting condition (control, social pressure, monetary incentive). The results of the analysis on *the proactive index for error rates* failed to show a significant effect of either condition, *F*_(2, 88)_ = 1.4, *p* = 0.243, starting condition, *F*_(2, 44)_ = 0.8, *p* = 0.437, or their interaction, *F*_(4, 88)_ = 0.5, *p* = 0.705 (see Figure 5A). Results therefore indicate a similar shift to proactive control regardless of which condition was presented first. The analysis of the *proactive index for reaction times* also failed to reveal a significant main effect of the condition, *F*_(1.73, 76.1)_ = 2.4, *p* = 0.103, starting condition, *F*_(2, 44)_ = 1.2, *p* = 0.323, or their interaction, *F*_(4, 88)_ = 1.1, *p* = 0.342 (see Figure 5B).

These results show that the order of experimental conditions does not affect the observed proactive control indices. We can therefore conclude that social pressure and instructions to perform better indeed independently increase proactive control rather than just continue the strategy adopted under the previous task condition. Overall, the results show an increase in the proactive control index in all three conditions, but it was least pronounced under the monetary incentive condition, irrespective of which incentive was presented first.

## Discussion

### Incentives improve performance beyond instructions

To enhance our understanding of what makes incentives effective, we need to examine how they affect the fundamental drivers of cognition. It is increasingly recognized that different incentives trigger distinctive cognitive coping strategies (Lerner and Tetlock, 1999; Vieider, 2011). Our goal was to understand how two prominent formal control mechanisms—social pressure and monetary incentives—affect cognitive control strategies to enhance performance beyond the effect of plain instructions to perform better. Our results indicate that instructing participants to improve their performance on its own leads to a significant shift toward proactive control and performance improvement compared to the baseline. Both monetary incentive and social pressure lead to further improvements in performance. The best performance in terms of the highest accuracy and the shortest reaction times is achieved under monetary incentive.

Our findings confirm prior results that monetary incentives improve cognitive performance, specifically by activating the proactive control mode, which enables participants to better update and preserve goal-relevant cue information throughout task performance (Locke and Braver, 2008; Jimura et al., 2010; Chiew and Braver, 2013, 2014; Fröber and Dreisbach, 2014). Interestingly, monetary incentive did not lead to the highest proactive control index, but to increases in both proactive and reactive control, an issue we specifically address below.

### Social incentives elicit proactive control and enhance performance

A specific contribution of our study to the existing research into factors influencing cognitive control is its examination of social incentives. The finding that social pressure also elicits proactive control and enhances performance contrasts with studies, which found that social anxiety and psychological pressure adversely affect performance (Hickman and Metz, 2015; Schmid et al., 2015). These opposing results may arise from a different approach: the study by Hickman and Metz (2015) is an event study investigating the psychological pressure invoked by high prizes in sports designed to elicit maximum performance. They found that such pressure negatively affects performance, but that less experienced athletes are more affected than their more experienced counterparts. Schmid et al. (2015) analyzed social anxiety as a personal trait rather than as a response to a socially threatening stimulus. For socially anxious people, compared to a healthy control group, such a stimulus represents a stronger conflict, which has to be processed by relying on reactive control. In cognitive tasks in which goal representation, maintenance, and information updates are required, the reactive mode leads to a worse performance than the proactive mode.

Pessoa (2009) explains that a low-threat stimulus enhances target processing as emotionally laden stimuli are prioritized. In contrast, a high-threat stimulus diverts cognitive resources toward processing the stimulus, which impairs performance. In the latter case, the focus is on monitoring a conflict and resolving interference, typical of reactive control. What also matters is the task relevance of a stimulus: a task-relevant stimulus improves performance as it directs more resources to the task, whereas a task-irrelevant stimulus impairs performance. Our findings indicate that social pressure may have been perceived as a low-threat, high-relevance stimulus by the participants. It is worth noting that Pessoa (2009) refers to stimulus-driven threat effects elicited by trial-by-trial manipulations, whereas our manipulation is block-based and gives rise to a sustained activity. The differences between transient and sustained effects will have to be analyzed in future studies as the present evidence in the literature does not systematically examine them.

### Instructions in the absence of specific incentives improve performance

Experimental studies tend to neglect the effect of the lab environment on participants. As revealed in our control condition, performing better when being asked to may in itself have a positive effect on performance through a natural inclination to obey authority (Milgram, 1974). In several of our analyses, we found similar effects between the two incentives and the control condition. This implies that, when participating in an experiment, mere instructions to improve performance have similar effects to instructions that announce social or monetary consequences. The studies that do not control for such a global effect of instructions might falsely attribute the entire effect to monetary incentives.

In contrast, the AX-CPT study by Chiew and Braver (2013, partly replicated in 2014) employed an experimental design comparable to ours. They directly compared block (sustained) reward manipulations (non-incentive trials within the reward block with non-incentive trials in the baseline condition) and trial (transient) reward manipulations (incentive to non-incentive trials within the reward block). Another valuable source of comparison is Fröber and Dreisbach (2014) who partly replicated the experimental design of Chiew and Braver (2013) with respect to performance-contingent reward manipulation. The results of all three studies are substantially similar to ours: they showed a shift to proactive control for non-incentive trials within the reward block compared to the non-incentive baseline block.

While the analysis of trial reward manipulation in the cited papers (Chiew and Braver, 2013, 2014; Fröber and Dreisbach, 2014) focused on the transient vs. the sustained effects of incentives, the non-incentive trials within the reward block could be affected by instructions that activated proactive control in the block. By this design, the authors managed to isolate the effect of incentives without confounding it with the effect of instructions, similar to our control and monetary incentive conditions, with the difference that their manipulation was transient while ours is sustained. This difference is not negligible as proactive control requires context representations to be sustained over extended periods, whereas in the reactive mode, the representation of the context is transient and maintained only when needed. Non-incentive trials in the reward block may thus be affected by a sustained proactive control mode, triggered by incentive trials. In our design, these two blocks are separate and there is less interference in terms of sustained cognitive control activity between one and the other, leading to clearer estimates of the instruction without incentive effect. All three studies found a further significant shift to the proactive mode for incentive trials (and significantly decreased accuracy for AY trials) when comparing behavioral performance in incentive and non-incentive trials within the reward block, a finding qualitatively similar to ours. Taken together, these results provide evidence that proactive control is already activated by instructions to perform better, and may be further intensified by incentives.

### Error penalty leads to balanced increase in both proactive and reactive control

The analysis of the proactive control index in our study lead to further insights. Whereas all three experimental conditions induced an increase in the proactive index compared to the baseline, direct comparison of the experimental conditions revealed no differences in the accuracy based proactive index and a decrease in the reaction time based proactive index in monetary incentive vs. both social pressure and control conditions. This seemingly contra intuitive finding needs to be understood in relation to overall performance in the experimental conditions, the relationship between proactive and reactive cognitive control, and the nature of the proactive control index itself.

The proactive control index, as computed in this and previous studies (e.g., Chiew and Braver, 2014), provides an estimate of relative reliance on proactive vs. reactive cognitive control, rather than an absolute measure of proactive control. If participants switch their cognitive control strategy from a reactive to a more proactive one, the index increases. If however, they rely on more reactive cognitive control, the index decreases. Though the proactive index computed in the AX-CPT implies that the two cognitive control mechanisms are antagonistic, DMC theory (Braver, 2012) postulates that proactive and reactive control involve potentially independent mechanisms that can be simultaneously engaged. Engagement of proactive control in the AX-CPT task enables faster and more accurate responses in trials in which the cue stimulus correctly predicts target response, but it also leads to slower responses and higher risk of errors in AY trials, in which the cue leads to incorrect anticipation of a relevant response. To ensure fast responses and low error rates in all conditions, the increase in proactive control needs to be complemented with an increase in reactive control, enabling swift and effective change in response in AY trials. The overall improvement in both reaction times and accuracy under monetary incentive compared to the other two conditions implies that monetary incentive leads to an increase in proactive but also reactive control: this is evident in the reduced ratio of proactive vs. reactive control reflected in the lower value of the proactive control index compared to the other experimental conditions.

Our results do not match the findings by Chiew and Braver (2014), which found a significant increase in the proactive index for error rates (but not for reaction times) in monetary incentive vs. non-incentive trials. This apparent discrepancy can, however, be ascribed to the differences in experimental design. In our study, participants were not only rewarded for fast and accurate responses, but also penalized for incorrect ones. This incentivized them to increase proactive control to enable faster responses, but also stimulated them to increase reactive control to avoid the penalty for increased errors in AY trials. Together this resulted in a smaller proactive control index compared to other experimental conditions with no error penalties. Chiew and Braver (2013, 2014), on the other hand, did not include a penalty for error responses in the incentive condition, allowing participants a more aggressive pursuit of the goal, relying primarily on an increase in proactive control without a concurrent increase in reactive control. Interpretation of our results is further supported by a functional magnetic resonance imaging study using the AX-CPT task in which Braver et al. (2009) found that penalty-based monetary incentives caused a shift from primarily cue-related to probe-related activation in a number of PFC regions of interest.

It could be argued that including a penalty in the monetary incentive condition prevents a direct comparison of the effectiveness of monetary incentives and social pressure in their ability to modulate cognitive control. We included a penalty into the monetary incentive condition because the aim of this paper is to contribute to practical implications. In organizational environments, the primary goal is not to stimulate proactive control, but to enhance performance, which includes appropriate protection from low-probability errors. Additionally, in the social pressure manipulation, we emphasized accuracy and speed (both mattered for ranking) and as suggested by Dambacher et al. (2011) the same is achieved with a penalty for errors, which also emphasizes accuracy rather than just speed. If we had designed monetary incentives with rewards only, the two conditions would have been less comparable. Our results suggest that only an explicit error penalty elicits concurrent increase in both proactive and reactive control and ensures the best overall performance.

### Monitoring as performance enhancer

As our results suggest, the key element in stimulating better performance is a combination of explicit instructions and monitoring, regardless of how monitoring relates to the ultimate monetary rewards or social evaluation. There is an important practical implication of this line of reasoning. For example, measuring performance is a costly venture for most firms, especially since such measurement should be objective to avoid negative social comparison side effects. Without the need for incentivizing or inducing pressures, simply observing working behavior may come at a lower cost and lower risk of potential dissatisfaction. Such positive effects can be expected especially when monitoring can be organized horizontally (Komaki, 1986; Towry, 2003).

However, when combined with performance non-contingent rewards, the instructions to perform better do not seem to trigger a more proactive mode of cognitive control. Fröber and Dreisbach (2014) compared cognitive strategies elicited by performance-contingent and performance non-contingent rewards. They showed that only the former shifts cognitive control to the proactive mode (faster reaction times and higher error rates in AY trials). In the performance non-contingent reward condition, reaction times did not differ from the baseline condition. In the reward manipulation coupled with a neutral emotional stimulus, participants made significantly fewer errors in AY trials, suggesting a shift toward a less proactive/more reactive mode. However, in the condition combined with a positive emotional stimulus, no effect on the cognitive control strategy was detected. The performance non-contingent reward scheme actually allowed the participants to perform worse and be rewarded for it but, interestingly, their performance did not deteriorate. They maintained the same performance as in the baseline condition, which may be an effect of intrinsic motivation and feedback.

Which type of incentives work best in an applied context depends on current task demands and available context information. If goal-oriented behavior is to be promoted, incentives that stimulate the proactive control mode are helpful. However, proactive control is not cost-free as evidenced by the increased error rates in the trials that require inhibition of a prepotent response. This might be especially important in an applied context where errors must be avoided at any cost because they have serious (possibly disastrous) consequences. For optimal cognitive performance in such tasks, the reactive mode is a better cognitive strategy as it is oriented to error detecting and conflict monitoring. Dreisbach (2006) and Fröber and Dreisbach (2014) found that a less proactive/more reactive mode was elicited by positive affect. Positive affect was found to positively influence cognitive flexibility (Isen et al., 1992; Ashby et al., 2002; Dreisbach, 2006) and creativity (Amabile et al., 1986), while a rigid imposition of incentives has been found to negatively impact creativity (Stanton, 2000; Bonner and Sprinkle, 2002; Frey and Osterloh, 2002). What further complicates the design of an appropriate management control system is the finding that the influence of positive affect may be easily overridden by performance contingent incentives (Fröber and Dreisbach, 2014).

### Limitations and further directions

The size of the observed effect of our monetary and non-monetary incentive conditions also importantly depends on the experimental design. Had we varied the intensity of each condition (i.e., a higher monetary reward, more distressing social incentive), their relative effects could have been different. It would be insightful to test varying intensities of the presently analyzed two conditions in future research. As the AX-CPT is a relatively simple task, whereas real-world tasks are more complex, variation in task difficulty could also lead to different relative outcomes. A generalizable finding, however, is that various incentive types are available in organizations to achieve improved cognitive performance and that their ultimate effect on performance depends on their relative intensity. We have shown that not only monetary incentives but also non-monetary incentives (ranking, instructions, monitoring) can be quite effective. These findings provide an important contribution concerning the effectiveness of various mechanisms within an organizational control system.

Other limitations of our study need to be considered when generalizing from our findings. By focusing on the comparison between social and monetary stimuli on cognitive performance, we did not measure social anxiety and reward sensitivity as personality traits. As found by Jimura et al. (2010), the strongest effects of monetary incentives on cognitive control and performance in a high load working memory task are observed in highly reward-sensitive individuals (an increase in both sustained and transient activation of the right DLPFC). If monetary incentive is coupled with trait reward sensitivity, the effect is reinforced.

It is much more difficult to predict the interaction between social pressure and trait social anxiety. A general finding in the literature is that trait anxiety is associated with increased reliance on reactive control, driven primarily by dACC activity (Schmid et al., 2015) and right ventrolateral prefrontal cortex (VLPFC) activity (Fales et al., 2008). As presented by Schmid et al. (2015), highly anxious people are particularly sensitive to socially threatening stimuli, but a low-anxiety group can also be driven to a reactive control mode by a threatening stimulus (Fales et al., 2008). To our knowledge, there is little prior evidence on how healthy individuals react to stimuli similar to the social pressure used in this study. Our participants generally shifted toward the proactive mode. Given the conflicting results in the literature finding both, the positive effect (Latham and Locke, 2006) and the “choking” effect of social pressure on performance, it might be that our participants were not particularly socially anxious. Low-anxiety individuals rely primarily on prefrontal cortex-mediated control processes in cognitive conflict tasks (Schmid et al., 2015). Low anxiety and increased activity in the DLPFC is also associated with approach motivation (Harmon-Jones, 2003).

To rule out that the sample in our study could be systematically skewed in personality traits resulting in participants being more responsive to one or another incentive, and to address some of the concerns that our results might be affected by trait anxiety, or any other personality trait which we did not measure, we investigated whether participants might be differentially affected by the incentive conditions used. The results showed quite the reverse: those who achieved high performance under the monetary incentive condition also performed well under social pressure and in the control condition, and vice versa. This further indicates that monetary incentives and social pressure have similar motivational effects. To be able to generalize the findings to a broader and possibly a more variable population, the moderating effect of personality traits certainly needs to be addressed in future studies. Nevertheless, we believe that the absence of personality trait analysis does not limit the validity of the comparison between stimuli-driven cognitive strategies: the sample is relatively homogenous (in terms of age, which minimizes age-related differences in cognitive control preferences) and the approach examines within-subject effects.

The next step in this line of research is to further validate the behavioral findings using neuroscientific methods. Research in cognitive neuroscience has already identified brain regions and networks implicated in response to monetary incentives or threat stimuli, and has examined their impact on cognitive control strategies (Braver et al., 2007; Fales et al., 2008). Since behavioral results demonstrate that social pressure and instructions shift cognitive control toward the proactive mode, we would expect to find sustained activity of the DLPFC similar to that elicited by monetary incentives, typical of the proactive control mode. In a trial-by-trial analysis, we would anticipate increased transient activity of the dACC and DLPFC in the baseline relative to all three conditions, in particular in trials that require response inhibition. Another relevant direction for future research may also entail testing other formal control mechanisms in order to compile a comprehensive picture of incentive effects on cognitive control. Greater understanding is needed about which control environments in practice represent informational cues handled by reactive and proactive control.

## Conclusion

Our study provided the first direct comparison of the effects of social pressure and monetary incentive on behavioral performance. Surprisingly, whereas the results showed the two incentive conditions to be comparable in their effect, much of the effect seems to be generated by the presence of explicit instructions to improve behavior and accompanying monitoring of behavior. The key advantage of monetary incentives, primarily ascribed to explicit error penalties, is ensuring that the increase in performance in most of the trials enabled by increased proactive control was not traded off by increased errors on those occasions that required effective reactive control.

The effect of incentives and other situational factors on cognitive control, effort, and performance will continue to offer a vast opportunity to advance our knowledge. Making use of brain imaging would allow us to identify the brain regions involved in processing pressure/incentives in cognitive control processes, which would provide important insights into the effect of incentives on human cognition. Making incentive systems work is vitally important for the overall functioning of organizations and society. In recent years, research has focused on the dysfunctional effects of these systems (Trotman et al., 2011), which may be a consequence of the way conflicts between incentives and natural (automatic) responses are resolved. Given the current debate in companies and society about the problems of increasing monetary incentives, our findings provide an avenue to start reconsidering the essential role of social pressure and monitoring in organizations, countering prevalent reliance on monetary incentives to enhance performance.

## Author contributions

ML, FH, GR, and SS significantly contributed to the concept and design of the work. ML substantially contributed to the acquisition, analysis, and interpretation of the data for the work. While ML, GR, and SS noticeably contributed to drafting the work, FH revised it critically for important intellectual content. All authors approved the final version and agree to be accountable for all aspects of the work in ensuring that questions related to the accuracy or integrity of any part of the work are appropriately investigated and resolved.

## Funding

The study was funded by Faculty of Economics, University of Ljubljana. Financial support was also provided by Slovenian Research Agency grant J7-6829 to Grega Repovš.

### Conflict of interest statement

The authors declare that the research was conducted in the absence of any commercial or financial relationships that could be construed as a potential conflict of interest.

## Footnotes

^1^As we computed reaction times for correct responses only, we excluded these subjects from reaction time analyses due to missing values. To keep the same set of subjects in all analyses, these subjects were also excluded from analyses of error rates. Due to a concern that the absence of correct responses in AY trials might reflect an extreme use of proactive control strategy, we repeated the error rate analyses including all subjects. The results remained stable with no change in the observed effects (both the significance and the sign of the effects remained the same). In the interest of clarity, we report only the results of subjects with complete data.

^2^Due to a concern that in some conditions (BX and BY) fast response times might reflect valid anticipatory responses, we repeated the analyses without excluding reaction times faster than 200 ms. This did not alter the results as neither the significance nor the sign of the effects changed.

^3^Responses where neither the letter ≫N≪ nor ≫M≪ was pressed. Subjects mistakenly pressed the letter B in eight cases.
