# Supplementary material for: The Impact of Social Pressure and Monetary Incentive on Cognitive Control
Source: Front Psychol. 2016 Feb 9;7:93. doi: 10.3389/fpsyg.2016.00093 (PMC4746438; doi:10.3389/fpsyg.2016.00093)
Supplement: Supplementary file 1 [file Presentation1.PDF]

Supplementary Material

The impact of social pressure and monetary incentive on cognitive control

Mina Ličen*, Frank Hartmann, Grega Repovš, Sergeja Slapničar

*** Correspondence:** Corresponding Author: mina.licen@ef.uni-lj.si

1 Supplementary Analysis

1.1 Practice effect across experimental conditions

Due to the within-subject block design of the study there is a potential concern that differences between blocks reflect practice effects rather than experimental manipulation. To address this concern we conducted a logistic regression of accuracy and a linear regression analysis of reaction times on a trial number in the experimental conditions. As the trials were manipulated within-subjects, the subjects were treated as a random factor and intercepts were modelled for each subject separately. Statistical significance was estimated using likelihood ratio χ^2 tests.

Results of a *logistic regression* of accuracy of responses (0 for an incorrect and 1 for a correct response) on a trial number did not reveal a significant effect of trials number in any condition (control condition, β < 0.002, $\chi^2(1) = 2.09$, $R^2 < .001$, $p = .148$, social pressure condition, β = -0.001, $\chi^2(1) = 0.40$, $R^2 < .001$, $p = .530$, monetary incentive condition, β = 0.001, $\chi^2(1) = 0.45$, $R^2 < .001$, $p = .501$; see **Supplementary Figure 1A**), suggesting that the accuracy did not improve with progression of the task. Results of a *linear regression* of reaction times on trial number also failed to show a significant effect in the control condition, β = -0.06, $\chi^2(1) = 1.66$, $R^2 = .18$, $p = .198$, and the monetary incentive condition, β = 0.03, $\chi^2(1) = 0.65$, $R^2 = .19$, $p = .422$, it did reveal a significant negative effect in the social pressure condition, β = -0.17, $\chi^2(1) = 16.87$, $R^2 = .17$, $p < .001$ (see **Supplementary Figure 1B**). Overall, results show no significant practice related improvements either on accuracy or reaction times in any of the experimental conditions, but rather a small effect of fatigue reflected in reaction times in the social pressure condition.

2 Supplementary Figures

2.1 Practice effect across experimental conditions

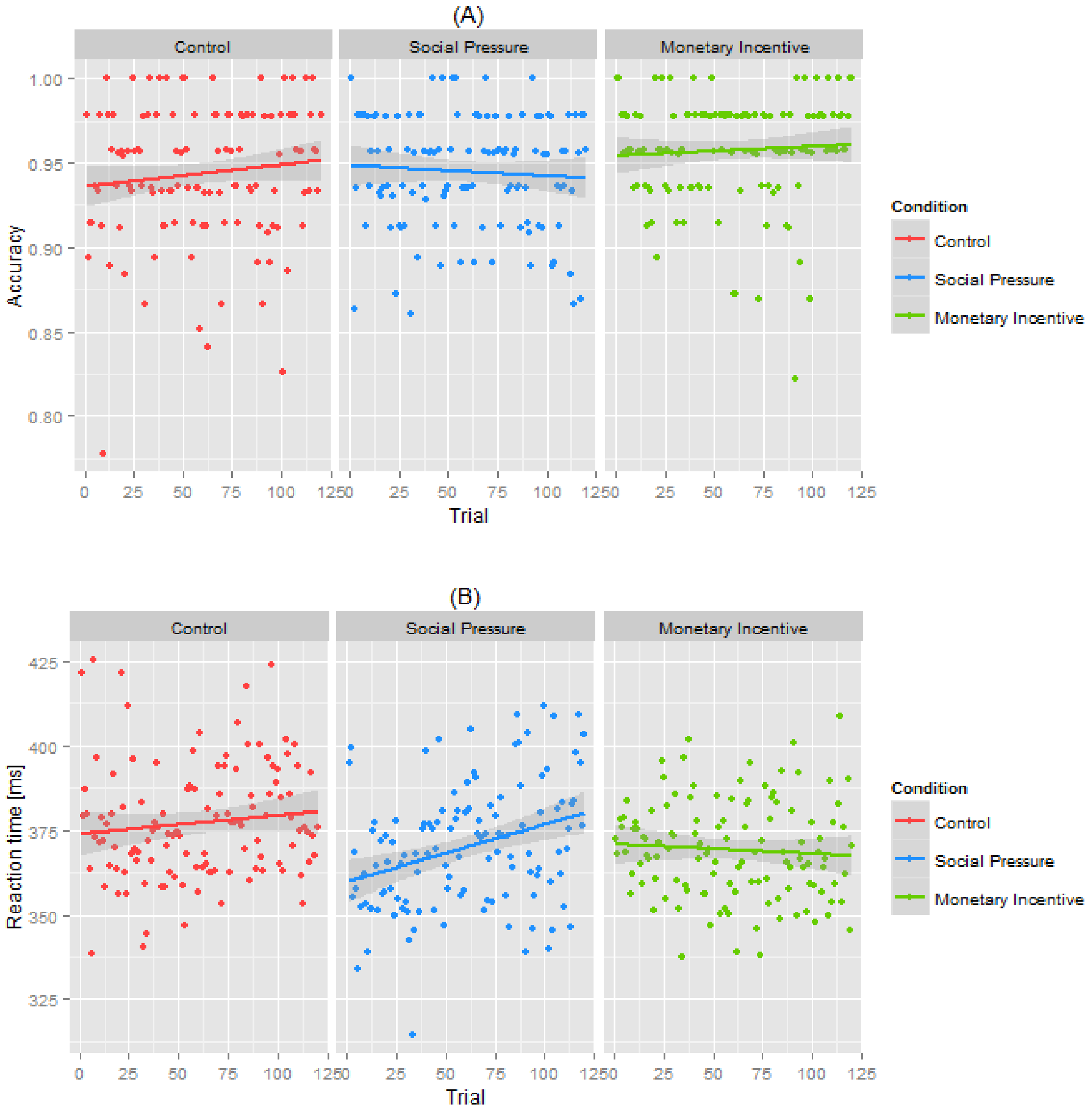

Supplementary Figure 1. Logistic regression of accuracy (A) and linear regression of reaction times (B) on trial number for each of the experimental conditions. A logistic regression of accuracy (A) and linear regression of reaction times of correct responses (B) collapsed over the trial type for all trials are shown in aggregated form for all the subjects in all the experimental conditions.

2.2 Speed-accuracy trade-off effect

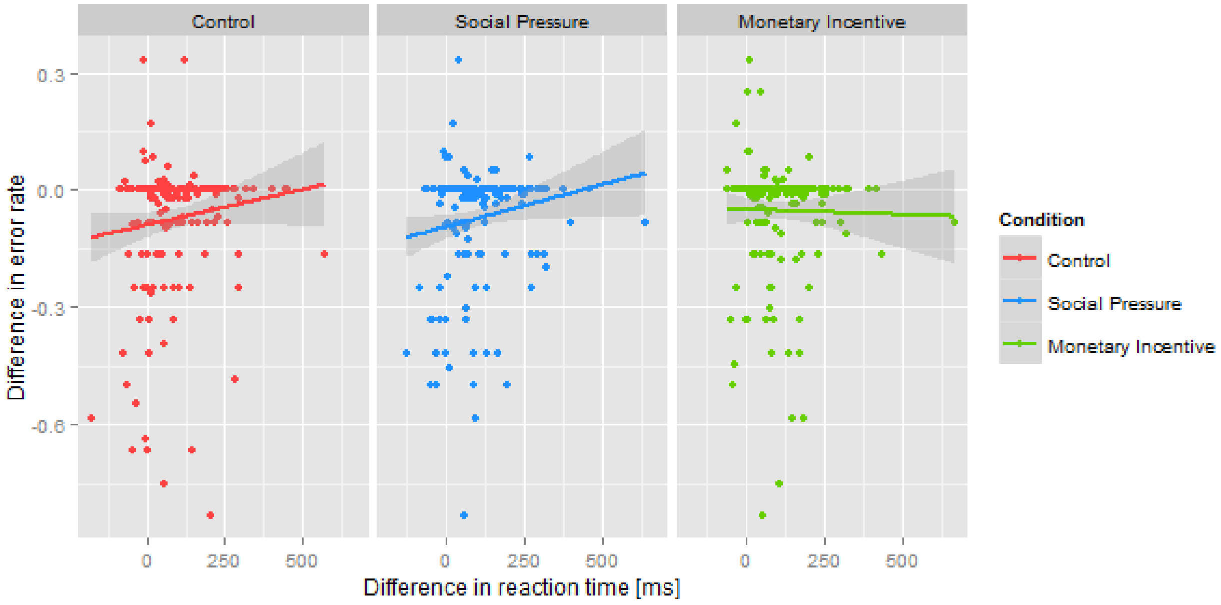

Supplementary Figure 2. Speed-accuracy trade-off plot for all experimental conditions. Changes in the error rates are plotted against changes in the mean reaction times of correct responses collapsed over the trial type for each of the incentive conditions. Differences in the error rates and reaction times are presented in milliseconds saved in a baseline vs. incentive condition. Positive values indicate an improvement in reaction times (shorter reaction time) and accuracy (lower error rate) under an incentive condition. A linear regression and its confidence interval are shown for each experimental condition.
